# High frequency of known copy number abnormalities and maternal duplication 15q11-q13 in patients with combined schizophrenia and epilepsy

**DOI:** 10.1186/1471-2350-12-154

**Published:** 2011-11-25

**Authors:** Larissa R Stewart, April L Hall, Sung-Hae L Kang, Chad A Shaw, Arthur L Beaudet

**Affiliations:** 1 Department of Molecular and Human Genetics, Baylor College of Medicine, Houston, TX 77030 USA

## Abstract

**Background:**

Many copy number variants (CNVs) are documented to be associated with neuropsychiatric disorders, including intellectual disability, autism, epilepsy, schizophrenia, and bipolar disorder. Chromosomal deletions of 1q21.1, 3q29, 15q13.3, 22q11.2, and *NRXN1 *and duplications of 15q11-q13 (maternal), 16p11, and 16p13.3 have the strongest association with schizophrenia. We hypothesized that cases with both schizophrenia and epilepsy would have a higher frequency of disease-associated CNVs and would represent an enriched sample for detection of other mutations associated with schizophrenia.

**Methods:**

We used array comparative genomic hybridization (CGH) to analyze 235 individuals with both schizophrenia and epilepsy, 80 with bipolar disorder and epilepsy, and 191 controls.

**Results:**

We detected 10 schizophrenia plus epilepsy cases in 235 (4.3%) with the above mentioned CNVs compared to 0 in 191 controls (p = 0.003). Other likely pathological findings in schizophrenia plus epilepsy cases included 1 deletion 16p13 and 1 duplication 7q11.23 for a total of 12/235 (5.1%) while a possibly pathogenic duplication of 22q11.2 was found in one control for a total of 1 in 191 (0.5%) controls (p = 0.008). The rate of abnormality in the schizophrenia plus epilepsy of 10/235 for the more definite CNVs compares to a rate of 75/7336 for these same CNVs in a series of unselected schizophrenia cases (p = 0.0004).

**Conclusion:**

We found a statistically significant increase in the frequency of CNVs known or likely to be associated with schizophrenia in individuals with both schizophrenia and epilepsy compared to controls. We found an overall 5.1% detection rate of likely pathological findings which is the highest frequency of such findings in a series of schizophrenia patients to date. This evidence suggests that the frequency of disease-associated CNVs in patients with both schizophrenia and epilepsy is significantly higher than for unselected schizophrenia.

## Background

The genetic contribution to the etiology of schizophrenia is significant, but the full molecular basis of the genetic factors remains incompletely defined. Since at least 1992 [1], it has been known that deletion of chromosome 22q11.2 is associated with schizophrenia with a very substantial relative risk. One study of 78 adults with deletion 22q11.2 found that 22.6% had schizophrenia [2]. Recurrent seizures were found in 39.7%, which may be explained in part by the hypocalcemia associated with this phenotype. In 2008, two studies [3,4] found that chromosomal deletions of 1q21.1, 15q13.3, and 22q11.2 were associated with markedly increased risk of schizophrenia; one report but not the other also suggested a role for deletion 15q11.2. These three most frequent deletions were found in 0.21%, 0.20%, and 0.26% respectively of the schizophrenic populations studied, and they are also associated with intellectual disability and autism [5,6]. It is quite remarkable that each of these CNVs can be associated with a wide range of phenotypes including intellectual disability, autism, schizophrenia, and epilepsy [7]. Deletion 15q13.3 is also reported to be present in as many as 1% of individuals with idiopathic generalized epilepsy [8]. For the 15q13.3 deletion, the *CHRNA7 *gene encoding the α7 subunit of the neuronal nicotinic receptor is a strong candidate gene mediating the phenotypic effects, as further supported by smaller deletions causing similar phenotypes [9]. The other two deletions (1q21.1 and 22q11.2) encompass many genes with no one gene yet strongly implicated as mediating the phenotypic effects. Other chromosomal mutations have also been found in schizophrenia such as deletions of 3q29 and *NRXN1 *and duplications of 15q11-q13 (maternal), 16p11.2, and 16p13.3 [10-14]. Deletions of chromosome 16p11.2 are particularly common in autism [15,16], and there is evidence that reciprocal duplications are associated with schizophrenia [11]. There is also evidence that loss-of-function mutations in individual genes can be associated with autism and/or schizophrenia. These genes include or may include *DISC1, NRXN1*, *PDE4B, NPAS3, CNTNAP2*, and *APBA2*; see Sebat et al. [7] for bibliography.

We reasoned that the frequency of detectable CNVs associated with schizophrenia might be higher in subjects with both schizophrenia and epilepsy compared to either phenotype alone. There is a report that patients with schizophrenia have an 11-fold increase in the prevalence of comorbid epilepsy [17]. We analyzed DNA from subjects with both schizophrenia and idiopathic epilepsy or with bipolar disorder and idiopathic epilepsy and from controls using array comparative genomic hybridization (CGH).

## Methods

### Subjects

DNA samples from 235 subjects with both schizophrenia and idiopathic epilepsy, 80 subjects with both bipolar disorder and idiopathic epilepsy, and 191 controls derived from cultured lymphoblasts, were obtained from the National Institute of Mental Health Human Genetics Initiative (NIMH-HGI). The NIMH website for this collection https://www.nimhgenetics.org/nimh_human_genetics_initiative/ reports that the establishment of a diagnosis of schizophrenia or bipolar disorder was based on DSM-III-R and DSM-IV criteria following a systematic and comprehensive examination of multiple sources of available information obtained from relatives, medical records, and direct assessment using the Diagnostic Interview for Genetic Studies for each patient. A self-reported questionnaire completed by the subjects was used to identify individuals who also had epilepsy in addition to a diagnosis of schizophrenia or bipolar disorder. Subjects reporting seizures secondary to causes such as trauma, medication, and/or polydypsia were excluded. Some patients with self-reported seizures but no further detail about etiology of their seizures were included. Ethnicity for the schizophrenia plus epilepsy samples was 135 Caucasian, 62 black, 22 Asian, 8 Hispanic, and 6 other. Ethnicity for the bipolar plus epilepsy was 77 Caucasian, 1 other, and 2 unknown. Ethnicity for the controls was 100% Caucasian. We screened Caucasian control individuals for psychiatric symptoms based on their self reported questionnaire. We chose to exclude controls with even modest or questionable behavioral findings in an attempt to exclude individuals who might have mild manifestations of a deleterious CNV. The rationale was to obtain a control group with under-representation of any behavioral abnormalities. Control individuals were excluded if they saw a doctor and took medication or used drugs and alcohol to deal with their depression or anxiety, if they reported any behaviors suggestive of obsessive compulsive disorder, or if they had a history of substance and/or alcohol abuse/dependence. We excluded 1284 samples out of 1920 leaving 636 control samples for study. Then 191 of the 636 were selected at random for study. This high exclusion rate was surprising, but it was already known that lifetime prevalences for depressive, anxiety, and substance use diagnoses were higher than in some other sample collections [18]. Additional information regarding screening criteria can be found in Additional file 1. Another study using these controls excluded far fewer samples based on rescreening [19].

### Array CGH

Blood collected from schizophrenia, bipolar disorder, and control samples was used to establish lymphoblastoid cell lines and DNA was extracted by Rutgers University Cell and DNA Repository, Piscataway, NJ. Blood DNA was not available for most samples, so it was not possible to test whether results were only in lymphoblast DNA and absent in blood DNA. All cases and controls were hybridized with the same Caucasian male reference DNA isolated from fresh blood. Although it is well known that using DNA from lymphoblast cell-lines can give rise to artifactual copy number changes [20,21], these usually do not produce CNVs identical to those known to cause neurobehavioral phenotypes and more often involve aneuploidy, very large genomic changes, or deletions at immunoglobulin genes.

Array CGH was performed on cases and controls with once-used, stripped clinical v8.0 arrays from the Medical Genetics Laboratory (MGL) at Baylor College of Medicine (BCM). Specifically, the v8.0 array is a 180 K Agilent oligonucleotide array with 30 kb backbone coverage and exon by exon coverage for 1714 genes reported to be associated with or cause disease or considered to be candidates for neurological disease association [22]http://www.bcm.edu/geneticlabs/test_detail.cfm?testcode=8655. The array was designed by Pawel Stankiewicz, March 2006 and manufactured by Agilent Technology (Santa Clara, CA). Previously used slides from both abnormal and normal cases were stripped by boiling in a 5 mM potassium phosphate buffer solution for 2 minutes. The reuse of clinical arrays provided a major cost reduction for these studies and allowed for comparison to a large body of clinical data using the same array design. We have experience that the used arrays detect known variants reliably if the Agilent DLR score is less than 0.30. These data indicate that false negative results would be rare. There is no significant concern regarding false positive results, because all CNVs were validated using new arrays with coverage suitable for the putative CNV.

The procedures for DNA digestion, labeling, and hybridization for the oligonucleotide arrays were performed according to the manufacturers' instructions, with minor modifications [23]. Slides were scanned into image files using the Agilent G2565 Microarray Scanner. Scanned images were quantified using Agilent Feature Extraction software (v10.7.3.), then analyzed for copy-number change using our in-house analysis package, as described previously [24-26]. All calls made with the in-house software were visually inspected to assess their validity. Additional information on analysis can be found in Additional file 1.

Common CNVs, CNVs located in introns or in non-genic regions, and calls less than one kb were noted in the analysis but are not reported here. Common calls, as defined by being seen more than 20 times in 20,000 cases analyzed by the Medical Genetics Laboratories at Baylor College of Medicine, were also not included. Array data has been deposited in the GEO database under accession number GSE23703. All coordinates are based on the Feb. 2009 UCSC Human Genome Browser assembly (hg19).

All calls reported were validated on a variety of fresh, higher resolution arrays, depending on the coverage of the region in question and included: a custom Agilent 1 M array designed to cover the great majority of exons in the genome (Celestion-Soper submitted manuscript), Agilent SurePrint G3 Human CGH 4 × 180 catalog array (design ID: 022060), and a gene targeted custom arrays (all Agilent Technologies, Santa Clara, California, USA). All arrays used in this study were designed and analyzed based on UCSC hg18 (NCBI Build 36), March 2006. For CNVs that did not have adequate coverage on the 4 × 180 Agilent catalog, custom Agilent 4 × 180 k or 8 × 60 k arrays with focused coverage for the regions of interest were designed using Agilent's E-array database. The array designs can be viewed on Agilent's e-array database https://earray.chem.agilent.com/earray/(SSC Tiling v2.0: 027305, SSC Focused v3.0: 028249, Focused v4.0: 028812).

CNVs found in our study were compared to those in the DGV database http://projects.tcag.ca/variation/. Entries in the DGV database that were smaller than or overlapped for less than 10% of our calls were not counted. IDENTICAL indicates one or more CNVs were found in the database to be identical to our CNV +or- 10% on either side. The publication by Levinson et al. [19] analyzed most of the same NIMH samples reported here. A tabulation of all CNV findings in individual samples reported here is available from the corresponding author.

## Results and Discussion

**Figure 1:**
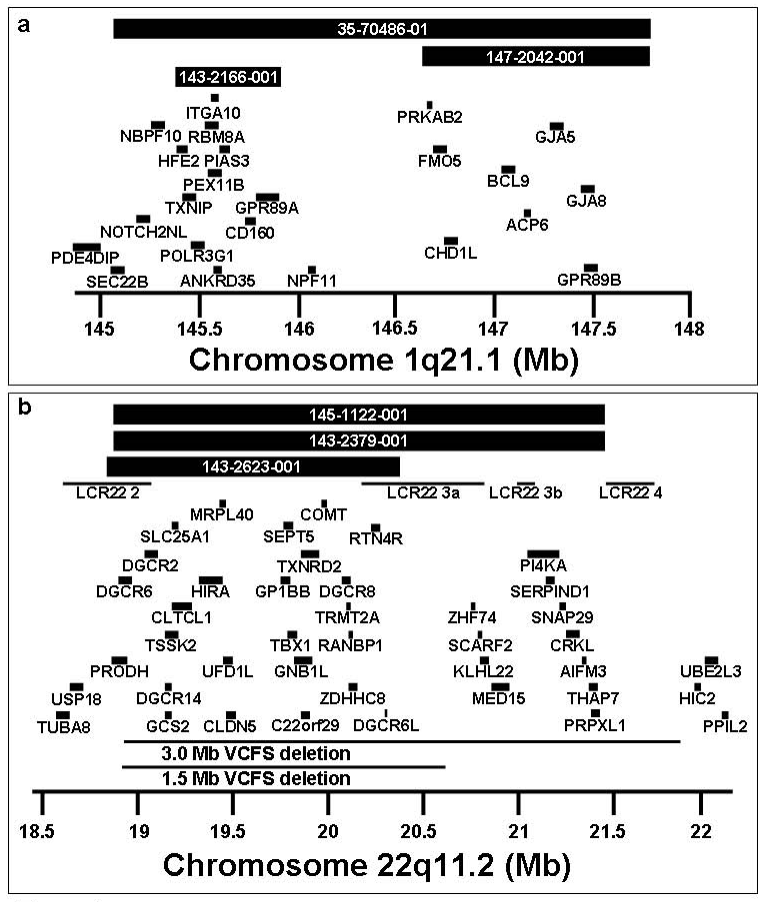
**Most frequent pathogenic microdeletions observed in NIMH cases with both schizophrenia and epilepsy**. Three cases of deletion 22q11.2 and three cases of deletion 1q21.1 are shown. Unique identifiers from the NIMH collection are provided. Low copy repeat (LCR) regions and gene symbols are provided. Coordinates are based on hg19.

DNA from 235 subjects with both schizophrenia and idiopathic epilepsy, 80 subjects with bipolar disorder and idiopathic epilepsy, and 191 controls was analyzed using array CGH. Out of 235 subjects with both schizophrenia and idiopathic epilepsy, we identified 10 CNVs well established to be associated with schizophrenia (see references in Background above), These included three deletions of 1q21.1, one deletion of 2p16.3 (*NRXN1*), one deletion of 15q13.3, two maternal duplications of 15q11-q13, and three deletions of 22q11.2 (Figure 1 and Table 1). We also found two other CNVs that have a well established role in other neuropsychiatric phenotypes or epilepsy. These included a deletion of 16p13.1 which is associated with intellectual disability and more recently with schizophrenia [27] and a duplication of the Williams-Bueren syndrome region (7q11.23) which is well documented to be associated with neuropsychiatric phenotypes [28]. In total we found 12 out of 235 cases of schizophrenia and epilepsy (5.1%) harbored CNVs highly likely to have a significant association with the disease phenotype. This is the highest detection rate for pathological CNVs in any schizophrenia population studied to date.

**Table 1:** CNV abnormalities of likely significance in cases with both schizophrenia and epilepsy.

*NIMH ID*	*Sex*	***Chr***.	*Start*	*Size (bp)*	*CNV*	*Genes*	*Validation*	*DGV*
35-70486-01	M	1q21.1	145009580	2814627	Loss	36	Cat	Identical
147-2042-001	M	1q21.1	146506369	1317838	Loss	15	Cat	Identical
143-2166-001	M	1q21.1	145388414	444581	Loss	17	Cat	Identical
147-2403-001	F	2p16.3	50579352	428671	Loss	*NRXN1*	Cat	Identical
143-2074-001	M	7q11.23	72876647	563297	Gain	15	Cus4	Identical
32-11055	F	15q11-q13	21213950	4994696	Gain	30 genes, SNORDs	Cat	Identical
144-1023-001	M	15q11-q13	22842145	6189545	Gain	28 genes, SNORDs	Cat	Identical
142-1340-001	M	15q13.3	30587903	2320279	Loss	16	MLPA	Identical
145-1243-001	M	16p13.11	15131782	1117825	Loss	10	Cat	Identical
145-1122-001	M	22q11.21	18894894	2569166	Loss	58	Cat	Identical
143-2379-001	M	22q11.21	18894894	2569166	Loss	58	Cat	Identical
143-2623-001	M	22q11.21	18890615	1469118	Loss	36	SNP	Identical

Abbreviations: M, male; F, female; Chr, chromosome; bp, base pair; CNV, copy number variant; DGV, Database of genomic variants. For validation: Cat = 4 × 180 Agilent catalog array; Cus1, Cus2, etc. refer to custom designed focused arrays 1, 2, etc.; MLPA-multiplex ligation-dependent probe amplification, SNP-Illumina 610 quad SNP catalog array.

**Figure 2:**
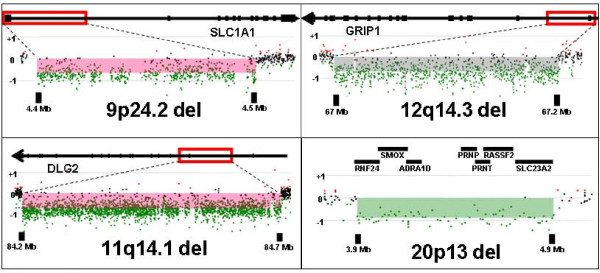
**Microdeletions of uncertain significance with some published link to neuropsychiatric disease observed in NIMH cases with both schizophrenia and epilepsy**. Deletions on specific Agilent custom arrays for validation are shown involving an exon of *DLG2*, an exon of *SLC1A1*, an exon of *GRIP1*, and multiple genes at the 20p13 locus as labeled. The regions of deletion are demarcated by red rectangles. Each dot represents an oligonucleotide, green for deleted and black for normal copy number.

Among cases with schizophrenia plus epilepsy, we identified many deletions of uncertain significance but with some published link to neuropsychiatric disease. Single cases of deletions were observed for 20p13, *CHRNB3*, *DLG2*, *GRIP1*, and *SLC1A1 *(Figure 2 and Table 2). A genome wide linkage analysis for autosomal dominant schizophrenia within a single Israeli Arab pedigree demonstrated a significant linkage association with 20p13 [29] and a linkage analysis of 270 Irish high-density families with varying psychotic illnesses showed a potential linkage of the behavioral phenotypes to a region on 20p that includes 20p13 [30]. There are multiple candidate genes in the linkage interval. *CHRNB3*, a nicotinic receptor, is reported to be associated with smoking behavior by GWAS [31] and to show linkage to epilepsy in one study [32]. *DLG2 *encodes a family member of the membrane-associated guanylate kinase (MAGUK) proteins which are part of the postsynaptic density and interact with receptors, ion channels, and other signaling proteins. Other members of the postsynaptic density such as PSD95 and SAP97 are reported to have altered expression in schizophrenia and epilepsy [33-35]. *GRIP1 *is a member of the glutamate receptor interacting proteins and has been reported to show altered expression in schizophrenia brain [36,37]. A study of genotyped autistic patients and matched controls showed an association of a SNP within a genomic region of *GRIP1 *with autism [38]. Linkage to the region containing, *SLC1A1*, a glutamate transporter, has been reported for obsessive compulsive disorder [39,40], and a SNP near SLC1A1 was reported to show association with autism spectrum disorder [41].

**Table 2:** CNVs abnormalities of possible significance in cases with both schizophrenia and epilepsy.

*NIMH ID*	*Sex*	***Chr***.	*Start*	*Size (bp)*	*CNV*	*Genes*	*Validation*	*DGV*
140-2082-001	M	8p11.21	42582092	7817	Loss	*CHRNB3*	Cus2	None
143-2447-001	M	9p24.2	4409405	134869	Loss	*SLC1A1*	Cus2	2 overlap
144-1472-001	M	11q14.1	84188228	482969	Loss	*DLG2*	Cus2	None
147-2093-001	M	12q14.3	67049795	150443	Loss	*GRIP1*	Cus4	None
144-1461-001	M	20p13	3897943	1039259	Loss	12	Cat	None

Abbreviations: As in Table 1.

Similarly, duplications of uncertain significance but with some published link to neuropsychiatric disease were found in five schizophrenia plus epilepsy cases. These included duplications of 6q15, 16p13.2, 19p13.3, *CHRNA7*, and *SLC6A4 *(Table 3). The 6q15 duplication contains *HTR1E *which encodes a serotonin receptor and has been suggested to be of interest in suicide [42], and attention-deficit/hyperactivity disorder [43]. The16p13.2 region contains ABAT which encodes a γ-aminobutyrate transaminase that is responsible for the conversion of GABA to succinic semialdehyde (OMIM 137150). This locus has been reported to show altered expression in epilepsy [44] and genetic association in autism [45]. There is some evidence for linkage to autism at 19p13.3 in a Finnish pedigree [46]. *CHRNA7 *small duplications are common in the population and may have some pathogenic significance [47], and at least one was reported in schizophrenia [3]. We identified two *CHRNA7 *duplications in schizophrenia patients and one in a bipolar disorder patient; however, we also found one in our controls. This duplication is quite common, but its phenotypic significance is not clear [47]. *SLC6A4 *encodes a protein involved in the transport of serotonin and there is a report of genetic association with schizophrenia [48]. Additional findings of unknown significance in schizophrenia plus epilepsy samples are listed in Table 3.

**Table 3:** CNVs of unknown significance in schizophrenia and epilepsy samples

*NIMH ID*	*Sex*	***Chr***.	*Start*	*Size (bp)*		*Genes*	*Validation*	*DGV*
142-1320-001	M	2p21	44531130	68210	Loss	*SLC3A1, PREPL, C3orf34*	Cus1	2 overlap
149-1129-001	F	2p16.1	56091146	13954	Gain	*EFEMP1*	Cus4	None
49-1020-001^a^	F	4q28.2	129774971	154534	Gain	*PHF17, SCLT1*	Cus4	Identical
53-10259	F	6p22.3	17465076	387198	Gain	4	Cus4	1 overlap
70-11763	F	6p22.3	22272906	148050	Loss	*PRL*	Cus4	1 overlap
144-1291-001	M	6q14.1	76607923	25447	Loss	*MYO6, IMPG1*	Cus1	None
54-10078	F	6q15	87456408	730692	Gain	9	Cus4	3 overlap
144-1096-001	M	7q11.22	71029067	240023	Gain	*WBSCR17, CALN1*	Cat	Identical
142-1006-001	F	7q31.32	122364506	676522	Gain	*CADPS2, TAS2R16, SLC13A1*	Cus4	5 overlap
149-1114-001	M	8q21	84569675	844484	Loss	*RALYL*	Cat	1 overlap
54-10151^a^	M	9p24.3	115980	471438	Loss	5	Cat	Common
141-0680-001	F	9p24.1	6451073	394797	Gain	*UHRF2, GLDC, KDM4C*	Cus4	4 overlap
142-1340-001	M	12p13.33	1949926	37541	Loss	*CACNA2D4*	Cus1	1 overlap
143-2467-001	M	12p13.33	1949882	34631	Loss	*CACNA2D4*	Cus1	1 overlap
146-2019-001	M	12p11.22	29009447	562052	Gain	*FAR2, ERGIC2*	Cat	1 overlap
142-1274-001	M	13q34	110957062	275718	Gain	*COL4A2, RAB20*	Cus4	1 overlap
140-2154-001	M	13q34	114476840	148441	Gain	*FLJ44054, GAS6, FAM70B*	Cus4	2 overlap
144-1307-001	M	15q11.2	22842143	244550	Gain	*TUBGCP5, CYFIP1, NIPA2,NIPA1 *^b^	MLPA	Common
147-2423-001	M	15q13.3	32296138	142658	Gain	*CHRNA7 *^b^	MLPA	Common
35-71412-02	M	15q13.3	32296138	164344	Gain	*CHRNA7 *^b^	MLPA	Common
42-1051-002	F	16p13.2	8969708	103443	Gain	USP7	Cat	1 overlap
143-2384-001	M	16p13.2	8581817	830333	Gain	9	Cat	1 overlap
147-2080-001	M	16p13.12	12771677	165576	Loss	*CPPED1*	Cat	2 overlap
54-10151^a^	M	17p13.3	2995336	422668	Gain	14	Cat	1 overlap
54-10151^a^	M	17p13.2	3743015	483613	Gain	8^b^	Cat	2 overlap
49-1020-001^a^	F	17p12	14111831	1330176	Gain	9	Cat	Identical
142-1278-001	M	17p12	14897946	148020	Loss	*CDRT7*	Cus4	Identical
144-1594-001	M	17p11.2	21311601	50888	Gain	*KCNJ12^b^*	Cus3	Identical
148-2171-001	M	17q11.2	28524944	21111	Gain	*SLC6A4*	Cus1	None
35-08447-02	M	18q11-q12	24744073	458607	Gain	*CHST9*	Cus4	None
30-11632	F	19p13.3	6427852	429890	Gain	14	Cus4	None
143-2526-001	M	19q13.42	54646013	1707820	Gain	87	Cat	7 overlap

Abbreviations: As in Table 1.

^a ^denotes samples having more than one CNV.

^b ^denotes cases which had a similar call to CNV found in controls.

For the 80 subjects with both bipolar disorder and epilepsy, only 13 CNVs were identified but the number of cases studied was relatively small. Bipolar disorder was previously reported to have a lower CNV detection rate as compared to schizophrenia [49]. The findings in patients with both bipolar disorder and epilepsy are listed and discussed in Additional file 1 Table S1.

The findings in the cases were compared to findings in the NIMH controls screened to reduce the frequency of psychiatric symptoms as discussed above. One control was found to have a duplication of 22q11.2. Duplications of 22q11.2 show incomplete penetrance and cause a highly variable phenotype of neurocognitive dysfunction [50,51], although there is at least one report suggesting an association with schizophrenia [10]. Although we screened out a large fraction of controls based on psychiatric symptoms, this individual passed that screen. Yet this male control with duplication 22q11.2 is noteworthy for self-reporting two episodes of depression lasting for four weeks accompanied by use of drugs or alcohol more than once for these problems. He also reported that drinking of alcohol interfered with school, job or home life once or twice in the past. He reported the use amphetamines, marijuana, and cocaine, and that this use interfered with school, job or home life 3-5 times. (We excluded controls reporting such drug use 6 or more times.) We also found a 15q11.2 deletion in one control sample, and though this CNV is reported to be associated with schizophrenia by some authors [3], the penetrance is quite low [12].

Other findings in controls involving loci of some neurobehavioral relevance included deletions of *CNTN4 *and *GABRR1 *and duplications of 15q11.2. Table 4 for complete list of CNVs found in controls.

**Table 4:** CNVs in psychiatric screened controls

*NIMH ID*	*Sex*	***Chr***.	*Start*	*size(bp)*		*Genes*	*Validation^a^*	*DGV*
150-10762	F	2p21	44549759	8313	Loss	*PREPL*	Cus4	None
150-11782	F	3p26.3	2695544	355319	Loss	*CNTN4*	Cus3	6 overlap
150-10112	M	3q29	196860921	478408	Gain	*DLG1, BDH1*	Cat	6 overlap
150-13123	M	6q15	89803425	144012	Loss	*SFRS13B, PM20D2, GABRR1*	Cus4	None
150-12782	M	5p13.2	37534374	305290	Gain	*WDR70, GDNF*	Cus3	None
150-12491	M	7p22.1	5667980	255505	Gain	*RNF216, ZNF815, OCM*	Cat	2 overlap
150-11643	F	7q21.13	88200226	354752	Loss	*ZNF804B, MGC26647*	Cus4	8 overlap
150-10750^a^	F	8p21.3	22211184	167245	Gain	*PIWIL2, SLC39A14, PPP3CC*	Cus4	2 overlap
150-12293	M	8q12.1	56682642	214806	Gain	*TMEM68, TGS1, LYN, RPS20*	Cat	4 overlap
150-12964	M	9q21.32	86522765	193620	Gain	5 genes	Cus4	3 overlap
150-10354	M	9q31.1	105642367	226151	Loss	*CYLC2*	Cus4	1 overlap
150-10750^a^	F	9q34.3	140374217	229402	Gain	9 genes	Cus4	None
150-12487	M	12q12	40789970	3711960	Gain	13 genes	Cat	None
150-12111^a^	M	12q24.12	112181037	136012	Gain	*ACAD10, ALDH2, C12orf47, MAPKAPK5*	Cus4	None
150-10678	F	12q24.21	116367980	170923	Gain	*MED13L*	Cus4	1 overlap
150-10629	M	13q33.1	103192748	327792	Gain	7 genes	Cat	None
150-12138	M	15q11.2	22842143	244550	Gain	*TUBGCP5, CYFIP1, NIPA2, NIPA1 *^b^	MLPA	Common
150-11886	M	15q11.2	22842143	244550	Gain	*TUBGCP5, CYFIP1, NIPA2, NIPA1 *^b^	Cat	Common
150-12900	M	15q11.2	22842143	244550	Loss	*TUBGCP5, CYFIP1, NIPA2, NIPA1*	MLPA	Common
150-11785	F	15q13.3	31730445	694760	Gain	*CHRNA7 *^c^	Cat	Common
150-11943	M	15q15.1	42656727	32418	Loss	*CAPN3*	Cus4	None
150-12111^a^	M	16p13.3	2147596	18151	Gain	*PKD1*	Cus4	3 overlap
150-12420	M	17p13.2	4027661	492396	Gain	10 genes^b^	Cat	4 overlap
150-10080	M	17p13.1	9993838	416496	Gain	5 genes	Cat	None
150-12837	M	17p11.2	21195549	306380	Gain	*MAP2K3, KCNJ12, C17orf51^b^*	Cat	13 overlap
150-12522	M	19q13.11	32809424	217110	Loss	*ZNF507, DPY19L3*	Cus4	None
150-10660	F	21q22	35720798	185606	Gain	*KCNE2, FAM165B, KCNE1, RCAN1*	Cus4	Identical
150-10685	M	22q11.21	18900180	2901481	Gain	50 genes	Cat	Common
150-10702	M	Xp22.12	19563240	384858	Gain	*SH3KBP1, CXorf23*	Cat	None
150-12093	M	Yp11.2	9523339	126272	Gain	8 genes	Cus4	None

Abbreviations: As in Table 1.

^a ^denotes samples having more than one CNV

^b ^denotes controls which had a similar call to CNV found in cases

We wished to assess whether the frequency of disease-related CNVs was significantly more frequent in patients with schizophrenia and epilepsy compared to controls from the same repository and tested on the same array platform. For this assessment, we accepted the following CNVs as being known to be associated with schizophrenia based on published data [12,19]: deletions of 1q21.1, 3q29, 15q13.3, 22q11.2, and *NRXN1 *and duplications of 15q11-q13 (maternal), 16p11, and 16p13.3. We included maternal duplications of 15q11-q13 in this group because of the extensive literature linking it to autism and to epilepsy and based on recent reports of its occurrence in schizophrenia [14,52]. We detected 10 schizophrenia plus epilepsy cases in 235 (4.3%) with the above mentioned CNVs compared to 0 in 191 controls (Fisher's exact test, p = 0.003). Other likely pathological findings in schizophrenia plus epilepsy cases included 1 deletion 16p13 and 1 duplication 7q11.23 for a total of 12/235 (5.1%) while a possibly pathogenic duplication of 22q11.2 was found in one control for a total of 1 in 191 (0.5%) controls (Fisher's exact test, p = 0.008). We also wished to assess whether our data supported the hypothesis that disease-associated CNVs would be more common in cases of schizophrenia and epilepsy compared to unselected schizophrenia. The rate of abnormality in the schizophrenia plus epilepsy of 10/235 for the more definite CNVs compares to a rate of 75/7336 based on supplemental data from Levinson et al. [19] for these same CNVs in a series of unselected schizophrenia cases (Fisher's exact test, p = 0.0004).

There are weaknesses in the current study including the use of cell line DNA rather than blood derived DNA, self-reporting of epilepsy findings, less than optimal phenotypic information, lack of availability of parental DNA, and lack of complete matching of ethnicity of cases and controls. Despite these difficulties, the observed differences are so substantial that they indicate that the frequency of disease-associated CNVs is significantly higher than any control group and significantly higher than in any series of unselected schizophrenia cases. Based on these results, clinicians can reasonably expect a pathological CNV detection rate of about 5% if they study patients with both schizophrenia and idiopathic epilepsy. In addition, all of these samples are readily available to diagnostic laboratories for validation of testing. These data, along with progress in whole exome and whole genome sequencing, suggest that the time is approaching when molecular genetic analysis of schizophrenia patients will be a clinically useful activity.

## Conclusions

The data presented here strongly suggest that disease-associated CNVs were present at a significantly higher frequency in cases with both schizophrenia and epilepsy compared to control samples from the same repository. In addition, disease-associated CNVs were found at a significantly higher frequency in schizophrenia plus epilepsy cases than in larger series of unselected schizophrenia. Although many geneticists would argue that chromosomal microarray analysis (CMA) is clinically useful today in patients with either schizophrenia or idiopathic epilepsy alone, the case for CMA testing is more compelling for individuals with both schizophrenia and epilepsy. The detection rate of clinically significant abnormalities is increasing with exon by exon coverage for hundreds of genes [22]. Although the frequency of pathological finding may be regarded by some as relatively low, definitive abnormalities can clarify diagnosis, inform genetic counseling, and potentially lead to CNV-specific management [53].

## Competing interests

All authors are based in the Department of Molecular and Human Genetics at Baylor College of Medicine (BCM), which offers extensive genetic laboratory testing, including use of arrays for genomic copy number analysis, and derives revenue from this activity.

## Authors' contributions

LRS and ALB designed the study. LRS and ALH performed experiments. S-HLK assisted LRS and ALH in interpretation of data. CAS provided statistical analysis. LRS and ALH drafted the manuscript. ALB supervised all activities and was responsible for the final version of the manuscript.

## Pre-publication history

The pre-publication history for this paper can be accessed here:

http://www.biomedcentral.com/1471-2350/12/154/prepub

## Supplementary Material

Additional file 1 **Supplementary**. Additional details regarding methods, data analysis, and validation of findings as well as other results not discussed in the text can be found here. Click here for file

## References

[B1] Shprintzen RJ Goldberg R Golding-Kushner KJ Marion RW Late-onset psychosis in the velo-cardio-facial syndrome Am J Med Genet 1992 42 141 142 10.1002/ajmg.1320420131 1308357

[B2] Bassett AS Chow EW Husted J Weksberg R Caluseriu O Webb GD Clinical features of 78 adults with 22q11 Deletion Syndrome Am J Med Genet A 2005 138 307 313 16208694 10.1002/ajmg.a.30984 PMC3127862

[B3] Stefansson H Rujescu D Cichon S Pietilainen OP Ingason A Steinberg S Large recurrent microdeletions associated with schizophrenia Nature 2008 455 232 236 10.1038/nature07229 18668039 PMC2687075

[B4] International Schizophrenia Consortium Rare chromosomal deletions and duplications increase risk of schizophrenia Nature 2008 455 237 241 10.1038/nature07239 18668038 PMC3912847

[B5] Niklasson L Rasmussen P Oskarsdottir S Gillberg C Autism, ADHD, mental retardation and behavior problems in 100 individuals with 22q11 deletion syndrome Res Dev Disabil 2009 30 763 773 10.1016/j.ridd.2008.10.007 19070990

[B6] Mefford HC Sharp AJ Baker C Itsara A Jiang Z Buysse K Recurrent Rearrangements of Chromosome 1q21.1 and Variable Pediatric Phenotypes N Engl J Med 2008 359 1685 1699 10.1056/NEJMoa0805384 18784092 PMC2703742

[B7] Sebat J Levy DL McCarthy SE Rare structural variants in schizophrenia: one disorder, multiple mutations; one mutation, multiple disorders Trends in Genetics 2009 25 528 535 10.1016/j.tig.2009.10.004 19883952 PMC3351381

[B8] Helbig I Mefford HC Sharp AJ Guipponi M Fichera M Franke A 15q13.3 microdeletions increase risk of idiopathic generalized epilepsy Nature Genet 2009 41 160 162 10.1038/ng.292 19136953 PMC3026630

[B9] Shinawi M Schaaf CP Bhatt SS Xia Z Patel A Cheung SW A small recurrent deletion within 15q13.3 is associated with a range of neurodevelopmental phenotypes Nat Genet 2009 41 1269 1271 10.1038/ng.481 19898479 PMC3158565

[B10] Rodriguez-Santiago B Brunet A Sobrino B Serra-Juhe C Flores R Armengol L Association of common copy number variants at the glutathione S-transferase genes and rare novel genomic changes with schizophrenia Mol Psychiatry 2010 15 1023 1033 10.1038/mp.2009.53 19528963

[B11] McCarthy SE Makarov V Kirov G Addington AM McClellan J Yoon S Microduplications of 16p11.2 are associated with schizophrenia Nature Genet 2009 41 1223 1227 10.1038/ng.474 19855392 PMC2951180

[B12] Vassos E Collier DA Holden S Patch C Rujescu D St CD Penetrance for copy number variants associated with schizophrenia Hum Mol Genet 2010 19 3477 3481 10.1093/hmg/ddq259 20587603

[B13] Mulle JG Dodd AF McGrath JA Wolyniec PS Mitchell AA Shetty AC Microdeletions of 3q29 confer high risk for schizophrenia Am J Hum Genet 2010 87 229 236 10.1016/j.ajhg.2010.07.013 20691406 PMC2917706

[B14] Ingason A Kirov G Giegling I Hansen T Isles AR Jakobsen KD Maternally derived microduplications at 15q11-q13: implication of imprinted genes in psychotic illness Am J Psychiatry 2011 168 408 417 10.1176/appi.ajp.2010.09111660 21324950 PMC3428917

[B15] Kumar RA KaraMohamed S Sudi J Conrad DF Brune C Badner JA Recurrent 16p11.2 microdeletions in autism Hum Mol Genet 2008 17 628 638 18156158 10.1093/hmg/ddm376

[B16] Weiss LA Shen Y Korn JM Arking DE Miller DT Fossdal R Association between microdeletion and microduplication at 16p11.2 and autism N Engl J Med 2008 358 667 675 10.1056/NEJMoa075974 18184952

[B17] Makikyro T Karvonen JT Hakko H Nieminen P Joukamaa M Isohanni M Comorbidity of hospital-treated psychiatric and physical disorders with special reference to schizophrenia: a 28 year follow-up of the 1966 northern Finland general population birth cohort Public Health 1998 112 221 228 9724944 10.1038/sj.ph.1900455

[B18] Sanders AR Levinson DF Duan J Dennis JM Li R Kendler KS The Internet-based MGS2 control sample: self report of mental illness Am J Psychiatry 2010 167 854 865 10.1176/appi.ajp.2010.09071050 20516154 PMC6385597

[B19] Levinson DF Duan J Oh S Wang K Sanders AR Shi J Copy number variants in schizophrenia: confirmation of five previous findings and new evidence for 3q29 microdeletions and VIPR2 duplications Am J Psychiatry 2011 168 302 316 10.1176/appi.ajp.2010.10060876 21285140 PMC4441324

[B20] Redon R Ishikawa S Fitch KR Feuk L Perry GH Andrews TD Global variation in copy number in the human genome Nature 2006 444 444 454 10.1038/nature05329 17122850 PMC2669898

[B21] Craddock N Hurles ME Cardin N Pearson RD Plagnol V Robson S Genome-wide association study of CNVs in 16, 000 cases of eight common diseases and 3, 000 shared controls Nature 2010 464 713 720 10.1038/nature08979 20360734 PMC2892339

[B22] Boone PM Bacino CA Shaw CA Eng PA Hixson PM Pursley AN Detection of clinically relevant exonic copy-number changes by array CGH Hum Mutat 2010 31 1326 1342 10.1002/humu.21360 20848651 PMC3158569

[B23] Ou Z Kang SH Shaw CA Carmack CE White LD Patel A Bacterial artificial chromosome-emulation oligonucleotide arrays for targeted clinical array-comparative genomic hybridization analyses Genet Med 2008 10 278 289 10.1097/GIM.0b013e31816b4420 18414211 PMC2782565

[B24] Shaw CJ Shaw CA Yu W Stankiewicz P White LD Beaudet AL Comparative genomic hybridisation using a proximal 17p BAC/PAC array detects rearrangements responsible for four genomic disorders J Med Genet 2004 41 113 119 10.1136/jmg.2003.012831 14757858 PMC1735660

[B25] Cheung SW Shaw CA Yu W Li J Ou Z Patel A Development and validation of a CGH microarray for clinical cytogenetic diagnosis Genet Med 2005 7 422 432 10.1097/01.GIM.0000170992.63691.32 16024975

[B26] Lu X Shaw CA Patel A Li J Cooper ML Wells WR Clinical implementation of chromosomal microarray analysis: summary of 2513 postnatal cases PLoS ONE 2007 2 e327 10.1371/journal.pone.0000327 17389918 PMC1828620

[B27] Ingason A Rujescu D Cichon S Sigurdsson E Sigmundsson T Pietilainen OP Copy number variations of chromosome 16p13.1 region associated with schizophrenia Mol Psychiatry 2011 16 17 25 10.1038/mp.2009.101 19786961 PMC3330746

[B28] Van der Aa N Rooms L Vandeweyer G van den Ende J Reyniers E Fichera M Fourteen new cases contribute to the characterization of the 7q11.23 microduplication syndrome European Journal of Medical Genetics 2009 52 94 100 10.1016/j.ejmg.2009.02.006 19249392

[B29] Teltsh O Kanyas K Karni O Levi A Korner M Ben-Asher E Genome-wide linkage scan, fine mapping, and haplotype analysis in a large, inbred, Arab Israeli pedigree suggest a schizophrenia susceptibility locus on chromosome 20p13 Am J Med Genet B Neuropsychiatr Genet 2008 147B 209 215 10.1002/ajmg.b.30591 17823922

[B30] Fanous AH Neale MC Webb BT Straub RE O'Neill FA Walsh D Novel linkage to chromosome 20p using latent classes of psychotic illness in 270 Irish high-density families Biol Psychiatry 2008 64 121 127 10.1016/j.biopsych.2007.11.023 18255048

[B31] Thorgeirsson TE Gudbjartsson DF Surakka I Vink JM Amin N Geller F Sequence variants at CHRNB3-CHRNA6 and CYP2A6 affect smoking behavior Nat Genet 2010 42 448 453 10.1038/ng.573 20418888 PMC3080600

[B32] Durner M Zhou G Fu D Abreu P Shinnar S Resor SR Evidence for linkage of adolescent-onset idiopathic generalized epilepsies to chromosome 8-and genetic heterogeneity Am J Hum Genet 1999 64 1411 1419 10.1086/302371 10205274 PMC1377879

[B33] Ohnuma T Kato H Arai H Faull RL McKenna PJ Emson PC Gene expression of PSD95 in prefrontal cortex and hippocampus in schizophrenia NeuroReport 2000 11 3133 3137 10.1097/00001756-200009280-00019 11043537

[B34] Kristiansen LV Beneyto M Haroutunian V Meador-Woodruff JH Changes in NMDA receptor subunits and interacting PSD proteins in dorsolateral prefrontal and anterior cingulate cortex indicate abnormal regional expression in schizophrenia Mol Psychiatry 2006 11 737 47 705 10.1038/sj.mp.4001844 16702973

[B35] Liu FY Wang XF Li MW Li JM Xi ZQ Luan GM Upregulated expression of postsynaptic density-93 and N-methyl-D-aspartate receptors subunits 2B mRNA in temporal lobe tissue of epilepsy Biochem Biophys Res Commun 2007 358 825 830 10.1016/j.bbrc.2007.05.010 17506987

[B36] Hammond JC McCullumsmith RE Funk AJ Haroutunian V Meador-Woodruff JH Evidence for abnormal forward trafficking of AMPA receptors in frontal cortex of elderly patients with schizophrenia Neuropsychopharmacology 2010 35 2110 2119 10.1038/npp.2010.87 20571483 PMC2922423

[B37] Sodhi MS Simmons M McCullumsmith R Haroutunian V Meador-Woodruff JH Glutamatergic Gene Expression Is Specifically Reduced in Thalamocortical Projecting Relay Neurons in Schizophrenia Biol Psychiatry 2011 10.1016/j.biopsych.2011.02.022 PMC3176961 21549355

[B38] Mejias R Adamczyk A Anggono V Niranjan T Thomas GM Sharma K Gain-of-function glutamate receptor interacting protein 1 variants alter GluA2 recycling and surface distribution in patients with autism Proc Natl Acad Sci USA 2011 108 4920 4925 10.1073/pnas.1102233108 21383172 PMC3064362

[B39] Walitza S Wendland JR Gruenblatt E Warnke A Sontag TA Tucha O Genetics of early-onset obsessive-compulsive disorder Eur Child Adolesc Psychiatry 2010 19 227 235 10.1007/s00787-010-0087-7 20213231

[B40] Samuels J Wang Y Riddle MA Greenberg BD Fyer AJ McCracken JT Comprehensive family-based association study of the glutamate transporter gene SLC1A1 in obsessive-compulsive disorder Am J Med Genet B Neuropsychiatr Genet 2011 156B 472 477 21445956 10.1002/ajmg.b.31184 PMC3082623

[B41] Kantojarvi K Onkamo P Vanhala R Alen R Hedman M Sajantila A Analysis of 9p24 and 11p12-13 regions in autism spectrum disorders: rs1340513 in the JMJD2C gene is associated with ASDs in Finnish sample Psychiatr Genet 2010 20 102 108 20410850 10.1097/YPG.0b013e32833a2080

[B42] Baca-Garcia E Vaquero-Lorenzo C Perez-Rodriguez MM Gratacos M Bayes M Santiago-Mozos R Nucleotide variation in central nervous system genes among male suicide attempters Am J Med Genet B Neuropsychiatr Genet 2010 153B 208 213 19455598 10.1002/ajmg.b.30975

[B43] Oades RD Lasky-Su J Christiansen H Faraone SV Sonuga-Barke EJ Banaschewski T The influence of serotonin- and other genes on impulsive behavioral aggression and cognitive impulsivity in children with attention-deficit/hyperactivity disorder (ADHD): Findings from a family-based association test (FBAT) analysis Behav Brain Funct 2008 4 48 62 10.1186/1744-9081-4-48 18937842 PMC2577091

[B44] Arion D Sabatini M Unger T Pastor J Alonso-Nanclares L Ballesteros-Yanez I Correlation of transcriptome profile with electrical activity in temporal lobe epilepsy Neurobiol Dis 2006 22 374 387 10.1016/j.nbd.2005.12.012 16480884

[B45] Barnby G Abbott A Sykes N Morris A Weeks DE Mott R Candidate-gene screening and association analysis at the autism-susceptibility locus on chromosome 16p: evidence of association at GRIN2A and ABAT Am J Hum Genet 2005 76 950 966 10.1086/430454 15830322 PMC1196454

[B46] Kilpinen H Ylisaukko-Oja T Rehnstrom K Gaal E Turunen JA Kempas E Linkage and linkage disequilibrium scan for autism loci in an extended pedigree from Finland Hum Mol Genet 2009 18 2912 2921 10.1093/hmg/ddp229 19454485 PMC2708134

[B47] Szafranski P Schaaf CP Person RE Gibson IB Xia Z Mahadevan S Structures and molecular mechanisms for common 15q13.3 microduplications involving CHRNA7: benign or pathological? Hum Mutat 2010 31 840 850 10.1002/humu.21284 20506139 PMC3162316

[B48] Vijayan NN Iwayama Y Koshy LV Natarajan C Nair C Allencherry PM Evidence of association of serotonin transporter gene polymorphisms with schizophrenia in a South Indian population J Hum Genet 2009 54 538 542 10.1038/jhg.2009.76 19713975

[B49] Grozeva D Kirov G Ivanov D Jones IR Jones L Green EK Rare copy number variants: a point of rarity in genetic risk for bipolar disorder and schizophrenia Arch Gen Psychiatry 2010 67 318 327 10.1001/archgenpsychiatry.2010.25 20368508 PMC4476027

[B50] Lo-Castro A Galasso C Cerminara C El-Malhany N Benedetti S Nardone AM Association of syndromic mental retardation and autism with 22q11.2 duplication Neuropediatrics 2009 40 137 140 10.1055/s-0029-1237724 20020400

[B51] Firth HV 22q11.2 Duplication GeneReviews http://www.ncbi.nlm.nih.gov/sites/GeneTests/review?db=GeneTests

[B52] Kirov G Grozeva D Norton N Ivanov D Mantripragada KK Holmans P Support for the involvement of large CNVs in the pathogenesis of schizophrenia Hum Mol Genet 2009 18 1497 1503 10.1093/hmg/ddp043 19181681 PMC2664144

[B53] Cubells JF Deoreo EH Harvey PD Garlow SJ Garber K Adam MP Pharmaco-genetically guided treatment of recurrent rage outbursts in an adult male with 15q13.3 deletion syndrome Am J Med Genet A 2011 155A 805 810 21594999 10.1002/ajmg.a.33917

