# Supplementary material for: High frequency of known copy number abnormalities and maternal duplication 15q11-q13 in patients with combined schizophrenia and epilepsy
Source: BMC Med Genet. 2011 Nov 25;12:154. doi: 10.1186/1471-2350-12-154 (PMC3239290; doi:10.1186/1471-2350-12-154)
Supplement: Additional file 1 — Supplementary. Additional details regarding methods, data analysis, and validation of findings as well as other results not discussed in the text can be found here. [file 1471-2350-12-154-S1.DOC]

**Additional Information**

High frequency of abnormalities in genomic copy number analysis in 235 cases of combined schizophrenia and epilepsy

Larissa Stewart*, April L. Hall*, Sung-Hae Lee Kang, Chad A. Shaw, Arthur L. Beaudet

Department of Molecular and Human Genetics

Baylor College of Medicine

Houston, TX 77030 USA

**Supplemental Methods**

**Control questionnaire**

The entire questionnaire completed by all individuals in the NIMH-HGI control group can be found at <https://www.nimhgenetics.org/documents/contol_screen_interview.pdf>. Individuals were ruled out for our study if the answered yes(1) to BOTH A8d and A8e, if they answered yes (1) to BOTH B14 and B15, if they answered (3), (4), or (5) to G2a, if they answered (5) to G7a, if they answered yes (1) to H3 AND (3), (4), or (5) to H3a, or if they answered anything other than 2 (no) or -2 (not asked) to any question in section I.

**Array CGH**

**Stripping Protocol**

Both abnormal and normal subjects were previously hybridized on these arrays. To strip the slides, they were boiled in a 5mM potassium phosphate buffer solution for 2 minutes. Stripped slides were scanned and evaluated looking at the cy3 and cy5 intensity values. Recommendations for Cy3 intensity was ~ 50 , while cy5 intensity was ~10. Slides were only re-used once after their initial use and quality control was assessed in the same manner as new slides (discussed below).This protocol was validated and is used by the Medical Genetics Laboratory (MGL) at Baylor College of Medicine. In order to confirm our findings, results from the once-used slides were validated on new slides to identify any false positive findings (discussed below).

**DNA digestion**

Briefly, 300ng-1ug of genomic DNA from samples and a male reference control was digested with AluI (5 units) and RsaI (5units) IU (Invitrogen, Carlsbad, California, USA or Promega, Fitchburg, Wisconsin, USA) for 2 h at 37C. The digestion was stopped by incubating the reactions at 65ºC for 20 minutes. The efficacy of the digestion was assessed by running 2uL of the digested DNA by agarose gel electrophoresis. DNA fragments ranging from 100 to 800 bps with most around 200bps was deemed a successful digestion.

**Fluorescent labeling of digested DNA**

The digested DNA was labeled with Bioprime Array CGH Genomic Labeling System following the manufacturer’s recommended protocol with minor additions (Invitrogen, Carlsbad, CA). Following the addition of random primers to the digested DNA, the mixture was boiled for 5 minutes then cooled on ice for 5 minutes. Cyanine 5-dCTP (for the experimental sample), cyanine 3-dCTP (for the reference sample) (PerkinElmer, Boston, MA), dCTP nucleotide mix, and exo-Klenow fragment (40 units/µL) were added and the reaction was incubated at 37°C for 2 hours then incubated at 65°C for 10 minutes to inactivate the enzyme. The labeled product was purified from unincorporated nucleotides using Millipore columns or plate (Millipore, Billerica, MA, USA). Briefly, for the columns: Tris-EDTA (TE) buffer was added to the samples which were then placed into vertical columns and spun at 8000 RCF for 10min, supernatant was discarded and additional TE added and spun at 8000 RCF for 10min. Finally the columns were transferred to a new tube, inverted, and spun for 1min to collect the product. For the 96 well plate, TE was added to the samples and added to the plate. The plate was then placed on a Millipore vacuum manifold for two 10 minute intervals interrupted by blotting of the plate, TE was then added again and the plate was placed on a shaker for 10min, lastly the product was pipetted from the plate wells.

# Hybridization of labeled DNA

# The nanodrop fluorospectrometer was used to measure DNA concentrantion and dye incorporation inorder to pair subjects with reference DNA according to the following criteria: pmoles of dye were required to be within 3 units and DNA concentration within 100ng/uL. For each hybridization, the labeled experimental and reference DNAs were boiled for 3 minuted and incubated at 37°C for 30-90 minutes with human Cot-1 DNA (Invitrogen, Carlsbad, CA), blocking agent and hybridization buffer (Agilent Technologies, Santa Clara, CA). The samples were then added to an array, which was placed inside a microarray hybridization chamber (Agilent Technologies, Santa Clara, CA), and allowed to hybridize at 65°C for 35-68 hours in a rotating hybridization oven (Agilent Technologies, Santa Clara, CA). Following hybridization, slides were washed following the manufacturer’s protocol (Agilent Technologies).

**Image and data analysis**

Microarrays were scanned into an image file using the Agilent G2565 DNA Microarray Scanner (Agilent Technologies, Santa Clara, CA) with 3 um resolution, and the images were quantified using the Agilent Feature Extraction software (version 10.7.3.1, Agilent Technologies). The FE software generates a quality control (QC) report for each array. The derivative log ratio (DLR) was used to assess the quality of the array where a high sore reflects a high probe-to-probe log ratio noise. Per Agilent, a DLR of 0.30 or below is considered an acceptable quality. Therefore, any array with a DLR above 0.30 was repeated. If the initial run utilized only 300-500ng of DNA, 1 ug of DNA was used in the subsequent run.

The resulting text files generated from the feature extraction software was imported into our in-house analysis program and into Agilent CGH-Genomic Workbench v5.0 software for analysis. Analysis of oligonucleotide CGH microarray data was by our in-house program was performed in 4 steps.  First, microarray feature extractions files were obtained from the Agilent Genomic Workbench scanning software per the manufacturer's protocol.  The feature extraction step includes analysis to remove spatial and intensity biases in the low level intensity data.  Next, a filtering procedure was used to flag low intensity features; the intensity filter is a mixture model analysis on the combined Cy3 and Cy5 intensity data.  Features with a combined Cy3 and Cy5 intensity value of more than 3 standard deviations below the mean of the high intensity mode were flagged and excluded from further analysis.  Segmentation of the remaining data was performed using a circular binary segmentation method with post processing to insure that regions had at least 3 genomic coordinate consecutive probes with the same sign of deviation in logRatio as well as median logRatio which exceeded 0.2 in absolute value on the log scale.   Calls in genomic backbone non-gene regions were suppressed from consideration unless they exceed 100kb in size. In addition, all calls made with the in-house software were visually inspected to determine their validity and later confirmed by a certified clinical cyto-geneticist. Cases were evaluated in order of arrival; the NIMH subjects were evaluated first and the controls last. However, the clinical cytogeneticist was blinded to the source of the calls. In the Agilent CGH-Analytics, statistically significant CNVs were determined using the aberration detection module (ADM)-2 algorithm with a threshold of 5.6 and a minimum of 5 consecutive probes with a minimum absolute average log2 ratio of 0.3 for any given region.

**Validation**

Validation of our notable findings, was carried out by a variety of arrays, depending on the coverage of the region in question and include; Agilent SurePrint G3 Human CGH 4x180 catalog array (design ID: 022060), 1 million exon by exon coverage custom array, and gene targeted custom arrays (all Agilent Technologies, Santa Clara, California, USA).

Agilent Catalog 4x180: This commercially available SurePrint G3 Human CGH Microarray 4x180K (Design ID: 022060, Agilent Technologies, Santa Clara, CA) contains ~180,000 60bp probes covering the entire genome with 13kb overall median probe spacing. This array was used to validate larger CNVs or CNVs in known disease regions.

Agilent 1x1million custom exon by exon array: This custom array was designed using Agilent Technologies e-array program. ~18,000 genes from the refSeq database (June 2008, hg18), which contained 273,832 exons, 3’ UTRs, and 5’ UTRs, were targeted for probe selection. All probes selected only mapped to one location in the genome, except for pseudo-autosomal regions on chromosome X and Y. For each exon, six probes were designed, three on the forward strand, and three on the reverse strand at the same coordinates. Probes were spaced and overlap was avoided. In cases where more than 6 probes per exon were available, probes with the highest quality scores were selected. If no probes within the exon were available, probes were selected from the neighboring introns.

Agilent 4x180k and 8x60k custom gene targeted arrays: For CNVs that did not have good coverage on the 4x180 Agilent catalog or the 1x1M exon-exon array, custom arrays with focused coverage for our regions of interest were designed using Agilent’s E-array database (https://earray.lchem.agilent.com/earray/). The v2.0 4x180k focused array contained focused probes for 10 regions, the v3.0 8x60kcontained focused probes for 15 regions and the v4.0 8x60k contained focused probes for 45 regions. The focused array was designed to have maximum coverage with non-overlapping high quality probes within the minimum call region from initial array. Coverage was also extended ~10kb to both sides of the minimum call region. Analysis of CNVs was done using Agilent’s Genomic Workbench (v5.0) with the following settings: aberration algorithm ADM-2, minimum of 3 consecutive probes per region, and a minimum absolute average log2 ratio of 0.25 for any given region.

**Supplemental Results**

In the schizophrenia plus epilepsy cases, we also detected deletions of unknown significance where we could find no published link or only a weak link to neuropsychiatric disorders including 2p21, 9p24.3, *CACNA2D4, CPPED1, MYO6*, *PRL*, *CDRT7*, and *RALYL*, although a few overlapping events have been reported or are found in databases. Homozygous deletion of 2p21 including three genes (*SLC3A1*, *PREPL*, and *C3orf34*) is known to cause hypotonia-cystinuria syndrome [1], with heterozygous parents presumably being normal. A deletion in 9p24.3 includes the *DOC8* gene and four others; a disruption of DOC8 was found in two unrelated patients with intellectual disability [2], but homozygous loss-of-function mutations of *DOC8* gene results in autosomal recessive hyper-IgE syndrome (OMIM 243700) with heterozygous parents being normal. The *CACNA2D4* gene [3] encodes an auxiliary calcium-channel subunit, and homozygous deficiency causes retinal cone dystrophy-4 (*RCD4*, OMIM 610478). *CPPED1* encodes a protein designated calcineurin-like phosphoesterase domain containg-1 that has yet to be implicated in disease. *FHIT* is a member of the histidine triad gene family and encodes dinucleosidetriphosphatase (OMIM 601153). *MYO6* encodes a protein involved intracellular vesicle and organelle transport, especially in the hair cell of the inner ear, and mutations have been associated with autosomal dominant and recessive hearing loss (OMIM 600970). *PRL* encodes prolactin, a peptide hormone associated with lactation. *CDRT7* is CMT1A duplicated region transcript 7, and its function is unknown. *RALYL* encodes an RNA binding protein that has not yet been linked to disease.

Finally, in the schizophrenia plus epilepsy cases, we detected duplications of unknown significance where we could find no published link or only a weak link to neuropsychiatric disorders including 6p22.3, 7q11.22, 7q31.32, 9p24.1, 12p11.22, 13q34 (2 non-overlapping), 17p12, 17p13.2, 17p13.3, 19q13.42, *CHST, EFEMP1, KCNJ12*, and *SCLT1,* although a few overlapping events have been reported or are found in databases (**Table 2**). The 6p22.3 duplication includes genes that are not associated with any neuropsychiatric phenotypes, but the dystrobrevin binding protein 1 (DTNBP1) gene is nearby and is the subject of positive and negative reports of linkage to schizophrenia [4,5]. The 7q11.22 duplication includes a Williams-Beuren syndrome region gene, *WBSCR17*, which encode an N-acetylgalactosaminyltransferase (Entrez Gene 64409), but is not located within the classic Williams syndrome critical region. The 17p13.3 duplication is distal to the Miller-Dieker syndrome (MDS) region and does not overlap the MDS critical region [6]. All other regions and genes listed have not been associated with neuropsychiatric disease.

Out of the 80 subjects with both bipolar disorder and epilepsy, only 13 CNVs were identified. Deletions observed that were previously reported in neuropsychiatric illness include: 3q29and 14q24.2-q24.3. The 3q29 deletion has been associated with intellectual disability and developmental delay [7] and with schizophrenia [8,9]. The 14q24.2-q24.3 deletion is quite large (3.2 Mb) and may be significant based on size alone; this region has been suggested to play a modifier role in schizophrenia [10]. Deletion of CACNB4 is not specifically linked to bipolar disorder, but heterozygous loss-of-function mutations cause episodic ataxia type 2 (OMIM 108500) [11], and there are rare reports of combined occurrence of schizophrenia with episodic ataxia 2 [12,13]. A 16p13.3 deletion, containing the genes *DNASE1* and *TRAP1,* was found, but neither gene has been suggested to be associated with neuropsychiatric disease.

A few duplications identified in our bipolar plus epilepsy cases have been implicated in bipolar and/or epilepsy, and include the loci 3q29, and 11q24.1 respectively. The 11q24.1 duplication has been linked to epilepsy [14]. 3q29 duplication syndrome has been characterized and is associated with mild to moderate MR [15]. Duplications of uncertain significance included; 3p26.3, 5q23.1, 8p22, and 8q13.2 (**Table S1**).

Table S1: CNVs in Bipolar Disorder and Epilepsy

| *NIMH ID* | *Sex* | *Chr.* | *Start* | *Size (bp)* |  | *Genes* | *Validation* | *DGV* |
| --- | --- | --- | --- | --- | --- | --- | --- | --- |
| 12-11227 | M | 2q23.3 | 152820874 | 73245 | Loss | *CACNB4* | Cat | Identical |
| 170-1234-001 | M | 3p26.3 | 949343 | 478176 | Gain | *CNTN6* | Cat | Identical |
| 171-1044-001^a^ | M | 3q29 | 193979180 | 1464954 | Loss | 15 | Cat | 3 overlap |
| 171-1044-001^a^ | M | 3q29 | 197398813 | 438177 | Gain | 8 | Cat | 6 overlap |
| 15-11220^b^ | F | 5q23.1 | 115222722 | 228863 | Gain | *AP3S1, AQPEP,COMMD10* | Cus4 | 2 overlap |
| 22-10345 | F | 8p23-p12 | 161470 | 35795059 | Loss | 250 | ARTIFACT |  |
| 15-10030 | F | 8p22 | 12688335 | 258882 | Gain | *C8orf79* | Cus4 | 2 overlap |
| 15-11220^a^ | F | 8q13.2 | 67925160 | 280256 | Gain | *COPS5,CSPP1,ARFGEF1* | Cus4 | 1 overlap |
| 20-11007 | F | 11q24.1 | 123464962 | 60284 | Gain | *GRAMD1B,SCN3B* | Cus1 | Identical |
| 177-1435-001^a^ | F | 14q24 | 72560410 | 3398685 | Loss | 49 | Cat | None |
| 11-12593 | M | 15q13.3 | 32311602 | 103183 | Gain | *CHRNA7*^b^ | MLPA | Common |
| 178-1237-001 | F | 16p13.3 | 3704199 | 17135 | Loss | *DNASE1,TRAP1* | Cus1 | None |
| 177-1435-001^a^ | F | 19q13.42 | 54632532 | 4323865 | Gain | 150 | ARTIFACT |  |

Abbreviations: As in Table 1; Artifact, CNV seen may be a cell line artifact.

^a^ denotes samples having more than one CNV
^b^ denotes cases which had a similar call to CNV found in controls
